# Diagnosing CAH-X syndrome by long-read sequencing and identifying a novel genotype

**DOI:** 10.1186/s13023-026-04356-9

**Published:** 2026-04-22

**Authors:** Zhen Li, Shiyi Xu, Qingxian Fu, Lingling Du, Qiuting Lin, Hongjie Lin, Huishu E., Hui Liu

**Affiliations:** Department of Endocrinology and Inborn Metabolic Diseases, Fujian Children’s Hospital (Fujian Branch of Shanghai Children’s Medical Center); College of Clinical Medicine for Obstetrics & Gynecology and Pediatrics, Fujian Medical University, Fuzhou, 350014 China

**Keywords:** Long-read sequencing, CAH-X syndrome, Ehlers-Danlos syndrome

## Abstract

**Background:**

To conduct long-read sequencing (LRS) testing for molecular diagnosis of CAH-X syndrome. This study collected clinical data and evaluated the phenotypes of Ehlers-Danlos syndrome (EDS) in 20 cases of 21-hydroxylase deficiency (21-OHD) children and performed genetic diagnosis for CAH-X syndrome by LRS.

**Results:**

Two of the 20 cases of pediatric patients with 21-OHD were ultimately diagnosed with CAH-X syndrome. Both patients were presented with concurrent phenotypes of EDS and 21-OHD, 1 of whom exhibited a novel CAH-X genotype resulting from large gene conversion between the *CYP21A2-TNXB* gene (chr6:32039426–32044184) and *CYP21A1P-TNXA* pseudogene, whereas the other patient carried a *TNXA/TNXB_CH-1* chimeric gene.

**Conclusions:**

This study identified a novel CAH-X genotype resulting from a large gene conversion and indicates that LRS may be a more reliable method for genetic diagnosis of CAH-X syndrome.

## Introduction

Congenital adrenal hyperplasia (CAH) is an autosomal recessive genetic disorder that results in a deficiency of the enzymes responsible for synthesizing adrenal corticosteroids. Approximately 95% of CAH cases are due to 21-hydroxylase deficiency (21-OHD) caused by defects in the *CYP21A2* gene [1]. The *TNXB* gene is closely linked to the *CYP21A2* gene and encodes the extracellular matrix glycoprotein tenascin-X (TNX). Defects in the *TNXB* gene may lead to Ehlers-Danlos syndrome (EDS), a hereditary connective tissue disorder characterized by joint hypermobility, skin hyperextensibility, and tissue fragility [2]. Further, generalized joint hypermobility, subluxations, chronic arthralgias, soft or velvety skin, mild skin hyperextensibility, and variable systemic manifestations are also reported, whose severity can be correlated to the dosage of dominant alleles [3].

Recent studies have found that approximately 10% to 15% of patients with 21-OHD have defects in the *CYP21A2* and *TNXB* genes, resulting in CAH-X syndrome (by the co-occurrence of both CAH and EDS phenotypes) [4–9].

**Fig. 1:**
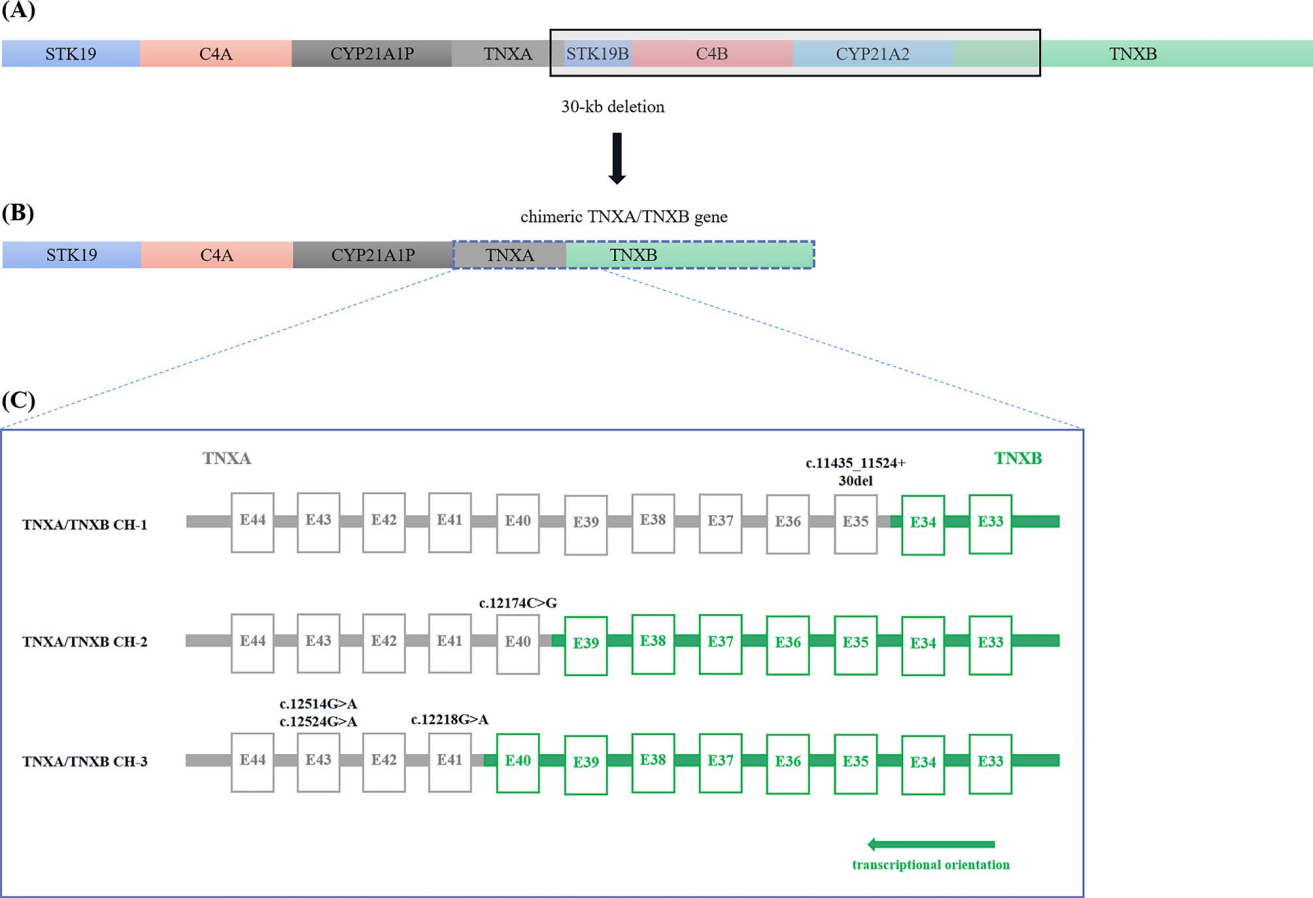
Schematic diagram of chimeric *TNXA/TNXB* gene. (**A**) Wild-type RCCX haplotype with bimodular structure. (**B**) The chimeric *TNXA/TNXB* gene in monomodular RCCX haplotype, caused by a 30-kb deletion. (**C**) Three types of chimeric TNXA/TNXB genes with different junction sites: CH-1, CH-2, and CH-3

Both *CYP21A2* and *TNXB* genes are located in the RCCX module of the major histocompatibility complex class III region on chromosome 6p21.33. The *CYP21A1P* pseudogene is located upstream of the *CYP21A2* gene that contains 10 exons and shares high homology with *CYP21A2*. Furthermore, the *TNXB* gene comprises 44 exons, whereas the *TNXA* pseudogene exhibits homology with exons 32 to 44 of *TNXB*. Compared with the *CYP21A2* and *CYP21A1P*, both *TNXB* and *TNXA* are transcribed in opposite directions, and their terminal exons overlap the 3ʹ untranslated region of *CYP21A2* and *CYP21A1P*, respectively. The genes *CYP21A2*, *CYP21A1P*, *TNXB*, and *TNXA* are arranged in tandem with the genes *STK19*, *STK19B*, *C4A*, and *C4B*, forming the RCCX module [10–12]. Highly homologous pseudogenes and functional genes in the RCCX module are prone to undergo recombination events during meiosis, with ~ 75% resulting in gene conversions and the remaining 25% leading to chimeric genes through homologous misalignment [12, 13]. Homologous misalignment may occur between *CYP21A2* and *CYP21A1P*, or between *TNXB* and *TNXA*, characterized by the formation of chimeric genes resulted because of the deletion of a ~ 30-kb gene sequence [11, 12, 14]. The *TNXB* gene and the *TNXA* pseudogene can undergo misalignment to form the chimeric *TNXA*/*TNXB* gene (CAH-X chimera). This chimera involves the deletion of a ~ 30-kb gene sequence that includes the entire *CYP21A2* gene and a part of the *TNXB* gene. As a result, both the *CYP21A2* and *TNXB* genes are impaired concurrently [14]. On the basis of the different junction sites, 3 types of chimeric *TNXA/TNXB* genes, that is, CAH-X CH-1, CAH-X CH-2, and CAH-X CH-3, have been reported. CAH-X CH-1 is characterized by the deletion of a 120-bp sequence (c.11435_11524 + 30del) that is derived from *TNXA* in exon 35; CAH-X CH-2 is characterized by the presence of a *TNXA*-derived variant c.12,174 C > G in exon 40; and CAH-X CH-3 is characterized by a cluster of multiple *TNXA*-derived variants especially from c.12218G > A in exon 41, c.12514G > A and c.12524G > A in exon 43 (Fig. 1) [4, 5, 15]. The CAH-X CH-1 chimera loses function because of the deletion of the 120-bp sequence, resulting in less expression through haploinsufficiency, whereas the expression levels of CAH-X CH-2 and CAH-X CH-3 chimeras remain unaffected but with alteration in protein structure associated with dominant negative effects [11, 12]. Additional studies reported that some patients with 21-OHD had no chimeric *TNXA/TNXB* gene but presented with manifestations of EDS, hinting at the possible existence of unknown CAH-X genotypes [7].

The molecular diagnosis of CAH-X syndrome possesses significant challenges, that is, the inability of conventional Sanger sequencing or next-generation sequencing (NGS) to detect CAH-X chimeras. Moreover, the detection of CAH-X chimeras typically relies on a specific PCR method that is not routinely performed in molecular diagnostic laboratories. In recent years, long-read sequencing (LRS) has been used for the molecular diagnosis of 21-OHD, which can simultaneously identify different types of variants, including single-nucleotide variations (SNVs), small insertions/deletions (indels), and chimeric genes, demonstrating promising detection capabilities [16–19]. LRS has emerged as a highly reliable method for diagnosing CAH-X syndrome due to its ability to resolve complex genetics, that is challenging for conventional techniques. Traditional methods such as Sanger sequencing and MLPA often struggle to distinguish between the functional *CYP21A2* gene and its pseudogene *CYP21A1P*, leading to diagnostic ambiguity. In contrast, LRS can span these repetitive regions in single reads, enabling accurate detection of gene conversions, large deletions, and chimeric rearrangements [20, 21]. A recent study by Wang et al. demonstrated that targeted LRS provided a more precise diagnosis in 14.6% of CAH cases compared to standard methods [20]. Similarly, Liu et al. showed that LRS-based assays could determine all genotypes of CAH with 100% sensitivity and specificity, outperforming MLPA and Sanger sequencing in resolving complex alleles and cis–trans configurations [17]. These findings highlight the clinical utility of LRS as a first-tier diagnostic tool for CAH-X. In this study, LRS testing was performed in 20 pediatric patients with 21-OHD to identify CAH-X syndrome; among them, 2 were genetically diagnosed with CAH-X syndrome, and 1 of them carried a novel genotype caused by a large gene conversion, expanding the genetic spectrum of CAH-X syndrome.

## Methods

### Patients

A total of 20 pediatric patients (7 boys and 13 girls, with an average age of 7.3 years) with 21-OHD who sought medical treatment at the Endocrinology Department of Fujian Children’s Hospital from September 2022 to September 2023 were included in the study. All patients were diagnosed as per the Endocrine Society Clinical Practice Guidelines (2018) [1]. All patients met the biochemical diagnostic criteria for 21-OHD, with a serum 17-hydroxyprogesterone (17-OHP) level exceeding 30 nmol/L, alongside clinical manifestations consistent with 21-OHD. This study was approved by the ethics committee of Fujian Children’s Hospital (No: 2022ETKLR10024), and informed consent was obtained from guardians of the patients.

### Evaluation of clinical phenotype

A retrospective review of clinical manifestations and laboratory tests related to 21-OHD at the onset of illness was conducted for each patient to classify them as salt-wasting, simple virilization (SV), or non-classical (NC) type. Further evaluation was performed to identify any patients who presented with EDS phenotypes. The Beighton 9-point scoring system [2, 22] was used to assess the joint hypermobility of patients, with a score of ≥ 5 indicating generalized joint hypermobility (GJH) in children [4]. Additionally, skin extensibility was evaluated using the following criteria: skin that can be stretched (a) > 1.5 cm on the distal forearms and dorsum of the hands or (b) > 3 cm on the neck, elbows, and knees, indicating skin hyperextensibility [2].

A physical examination was conducted to assess for additional signs related to EDS, including soft velvety skin, atrophic scars, unexplained skin striae, uvula bifida, high-arched palate, pes planus, and piezogenic papules. Additionally, a comprehensive review of medical history was conducted to ascertain the symptoms related to EDS, such as chronic joint pain, chronic muscle pain, joint laxity, recurrent joint dislocations, fragile skin, easy bruising, and delayed wound healing. Echocardiography was performed to examine for any cardiac defects in patients suspected of EDS manifestations.

### Detection of CAH-X genotype using long-read sequencing

Genomic DNA was prepared from peripheral blood samples using QIAamp DNA blood mini kit (QIAGEN) from the peripheral blood samples collected in EDTA tubes, quantified using a Qubit dsDNA BR assay kit (ThermoFisher Scientific), and detected using Qubit 2.0 fluorometer (ThermoFisher Scientific).

Two upstream primers, *A1P*-F and *A2*-F, and two downstream primers *TNXA*-R and *TNXB*-R, were designed. The primers *A1P-F/TNXA-R* were used to amplify the pseudogene *CYP21A1P-TNXA*, whereas *A2-F/TNXB-R* primers were used to amplify the functional gene CYP21A2-TNXB. Further, *A1P-F/TNXB-R* and *A2-F/TNXA-R* primers were used to amplify different chimeric genes, which resulted from homologous misalignments, including *TNXA/TNXB* chimeras. DNA samples were subjected to multiplex long-read PCR (LR-PCR) using KOD FX Neo (TOYOBO). PCR cycling conditions for optimal fragment amplification were 94 °C for 2 min (1 cycle); 98 °C for 10 s and 68 °C for 10 min (30 cycles), and 68 °C for 10 min (1 cycle). The PCR products were purified using 1×Ampure PB beads (Pacific Biosciences), quantified using Qubit dsDNA BR assay kit, and confirmed the successful amplification by agarose gel electrophoresis. For each purified PCR product, a single-step end repair and ligation reaction was performed to add a unique barcoded adapter. The 10 µl of reaction mix contained 4 µL of PCR product, 5 µmol/L barcoded adaptor (Integrated DNA Technologies), 1× T4 DNA ligase buffer (Enzymatics), 1 mmol/L ATP (New England Biolabs), 200 µmol/L dNTP (New England Biolabs), 2.5 units of T4 polynucleotide kinase (Enzymatics), 0.75 units of T4 DNA polymerase (Enzymatics) and 180 units of T4 DNA ligase (HC; Enzymatics). Reaction mixes were incubated at 37 °C for 20 min, 25 °C for 15 min, and 65 °C for 10 min. The failed ligation products were removed using exonuclease I (Enzymatics) and exonuclease III (Enzymatics). The pre-library was purified with 0.6×Ampure PB beads and quantified using Qubit dsDNA HS assay kit (ThermoFisher Scientific). Equal masses of uniquely barcoded pre-libraries were pooled together, purified using 0.6×Ampure PB beads, and quantified using a Qubit dsDNA HS assay kit. The single-molecule real-time (SMRT) bell library was prepared using Sequel Binding Kit 2.0 and Internal Control Kit 1.0 (Pacific Biosciences). Primed DNA-polymerase complexes were loaded onto SMRT cells and sequenced using Sequel II Sequencing Kit 2.0 on the Sequel II platform (Pacific Biosciences).

The circular consensus sequencing (CCS) software (Pacific Biosciences) was used to create CCS reads from the raw subreads in the BAM file output from the PacBio Sequel II platform. Furthermore, reads obtained from CCS were demultiplexed, and the barcode sequences were clipped using lima in the Pbbioconda package (Pacific Biosciences). Two reference sequences were created that is, the first reference sequence, *CYP21A2-TNXB*, contained within the genome sequence hg38 chr6:32037179–32,046,548, and the second reference sequence, *CYP21A1P-TNXA*, contained within the genome sequence hg38 chr6:32004843–32,013,648. The reference build *CYP21A1P-TNXA* was aligned with the reference build *CYP21A2-TNXB* using pbmn2, and FreeBayes1.3.4 was used to identify *CYP21A1P-TNXA* signature SNVs/indels. The junction site of chimeric genes resulting from large fragment deletion or conversion events was determined by the distribution of *CYP21A1P-TNXA* signature SNVs/indels in the reads. FreeBayes 1.3.4 was used for identifying SNVs/indels, and haplotype analysis was performed using WhatsHap. The pathogenic variants were displayed and confirmed in the Integrative Genomics Viewer (IGV) program with BAM files. (The specific LRS method was followed from the literature [17].)

## Results

Among the 20 pediatric patients, 2 patients (P1 and P2) were identified with characteristic clinical manifestations of EDS, with Beighton scores ≥ 5, and confirmed pathogenic variants in the *TNXB* gene by LRS testing. These 2 patients were ultimately diagnosed with CAH-X syndrome. Additionally, another 2 patients (P3 and P4) were reported to have a variant of uncertain significance and a variant of likely benign in the TNXB gene, respectively. However, no symptoms or signs associated with EDS were reported, with both having a Beighton score of 0. Therefore, they were not diagnosed with CAH-X syndrome. None of the remaining 16 patients had Beighton scores exceeding 3 (average score: 1.0) or were identified with pathogenic variants in the *TNXB* gene. A few children were found to have trifling skin symptoms (fragile skin or easy bruising) with no diagnostic significance. The clinical data and genetic testing results of these 20 patients are summarized in Table 1, and the case reports of the 2 patients with CAH-X syndrome are presented as follows.

**Table 1:** Clinical data and genetic results of 20 patients

Patient	Gender	Age (years)	21-OHD Pheno-type	EDS Phenotype		LRS results	
				Beighton score	Symptoms/signs related to EDS	CYP21A2-TNXB allele 1	CYP21A2-TNXB allele 2
1	Female	1	SV	6	Joint laxity, soft velvety and fragile skin, easy bruising, and borderline skin hyperextensibility	Large gene conversion (from the CYP21A2 exon 4 to the TNXB intron 33, as chr6:32039426–32044184)	CYP21A2:c.188 A > T,c.208G > T
2	Female	9	SV	5	Joint laxity	CYP21A2:c.518T > A	TNXA/TNXB_CH-1
3^**a**^	Female	11	SV	0	No findings	TNXB: c.12524G > A^**a**^; CYP21A2:c.955 C > T,c.1069 C > T	CYP21A2:c.518T > A
4^**b**^	Female	2	SW	0	No findings	TNXB: c.11,155 C > T^**b**^; CYP21A2:c.293–13 C > G	CYP21A2:c.293–13 C > G
5	Female	15	SV	3	No findings	CYP21A2:c.208G > T,c.188 A > T	CYP21A2:c.518T > A
6	Male	3	SW	2	No findings	CYP21A2:c.955 C > T, c.1069 C > T,c.*13G > A	CYP21A2:c.293–13 C > G
7	Female	8	SW	2	No findings	CYP21A1P/CYP21A2_CH-1	CYP21A2:c.293–13 C > G
8	Female	8	SV	2	No findings	CYP21A2:c.293–13 C > G	CYP21A2:c.1070G > A
9	Male	1	SW	2	No findings	CYP21A1P/CYP21A2_CH-8	CYP21A2:c.293–13 C > G
10	Female	5	SV	2	No findings	CYP21A2:c.293–13 C > G	CYP21A2:c.1280G > A
11	Male	3	SW	1	Easy bruising	CYP21A1P/CYP21A2_CH-3	CYP21A1P/CYP21A2_CH-1
12	Female	14	SV	1	No findings	CYP21A2:c.293–13 C > G	CYP21A2:c.293–13 C > G
13	Male	6	SV	1	No findings	CYP21A2:c.955 C > T, c.1069 C > T,c.*13G > A	CYP21A2:c.518T > A
14	Male	5	SW	0	Easy bruising	CYP21A1P/CYP21A2_CH-5	CYP21A1P/CYP21A2_CH-9
15	Female	18	SV	0	Fragile skin	CYP21A2:c.710T > A, c.713T > A,c.719T > A	CYP21A2:c.518T > A
16	Female	11	SV	0	No findings	CYP21A2:c.293–13 C > G	CYP21A2:c.518T > A,c.*13G > A
17	Female	14	SV	0	No findings	CYP21A2:c.923dup, c.955 C > T,c.1069 C > T,c.*13G > A	CYP21A1P/CYP21A2_CH-1
18	Female	8	SV	0	No findings	CYP21A2:c.293–13 C > G,c.*13G > A	CYP21A2:c.1280G > A
19	Male	2	SW	0	No findings	CYP21A1P/CYP21A2_CH-1	CYP21A2:c.292 + 1G > A
20	Male	7	SV	0	No findings	CYP21A2:c.332_339del	CYP21A2:c.518T > A

^**a**^In Patient 3, a c.12524G > A variant was detected in the TNXB gene, which is classified as a variant of uncertain significance (VUS) according to the ACMG guidelines^**b**^In Patient 4, a c.11,155 C > T variant was found in the TNXB gene, which is considered likely benign (LB) according to the ACMG guidelinesDue to the insufficient pathogenicity of the TNXB gene variants and the absence of any EDS-related phenotypes, Patients 3 and Patient 4 were not diagnosed with CAH-X syndrome

### Patient 1

A 1-year-old female toddler with elevated 17-OHP in serum was detected through newborn screening, without manifestations of salt-wasting such as feeding difficulties, vomiting, or fatigue. During the initial consultation, hyperpigmentation and mild clitoral enlargement in her external genitalia were reported. Before treatment, laboratory tests revealed significantly elevated levels of serum 17-OHP, adrenocorticotropic hormone (ACTH), and testosterone (T), with normal levels of serum sodium and potassium. Consequently, she was diagnosed with SV-type 21-OHD.

**Fig. 2:**
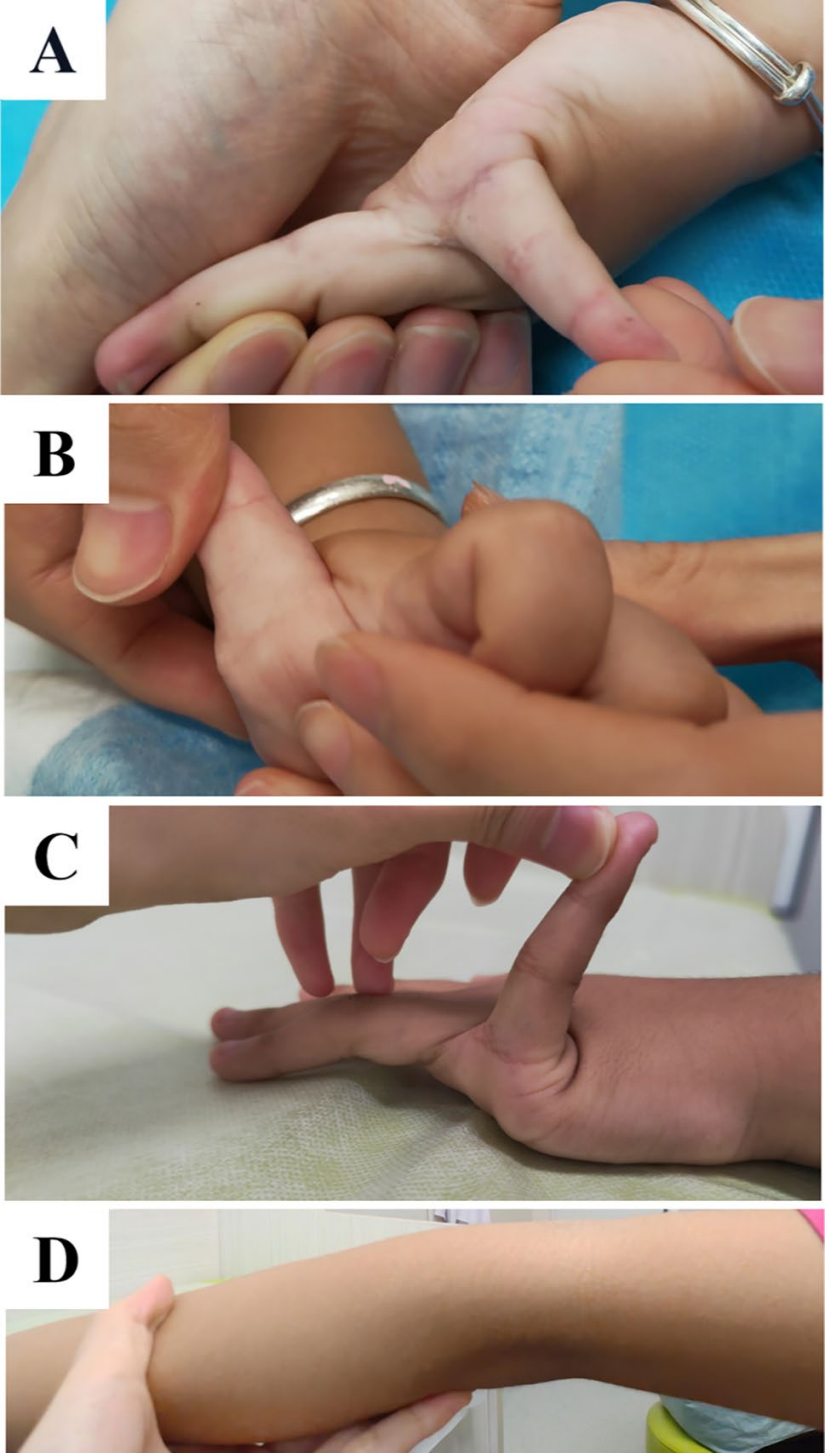
Joint hypermobility was found in 2 patients with CAH-X syndrome. (**A**) Positive for hyperextension of the fifth finger in patient 1. (**B**) Positive for thumb passively touching the ipsilateral forearm in patient 1 (**C**) Positive for hyperextension of the fifth finger in patient 2 (**D**) Positive for elbow hyperextension in patient 2

EDS phenotype assessment was conducted along with an inquiry into medical history. The assessments revealed fragile skin, susceptibility to bruising, and joint laxity. Physical examination revealed soft skin with a velvety texture. Her Beighton score was at least 6, indicating GJH with bilateral fifth finger metacarpophalangeal joints that could be passively hyperextended > 90° (Fig. 2A), bilateral elbow joints could hyperextend > 10°, and bilateral thumbs could passively touch the ipsilateral forearm (Fig. 2B), but the palm-to-floor test was not performed because of her young age. The skin on the distal forearm and dorsum of the hand could be stretched ~ 1.5 cm, suggesting borderline skin hyperextensibility. The results of the cardiac ultrasound examination did not reveal any cardiac defects.

**Fig. 3:**
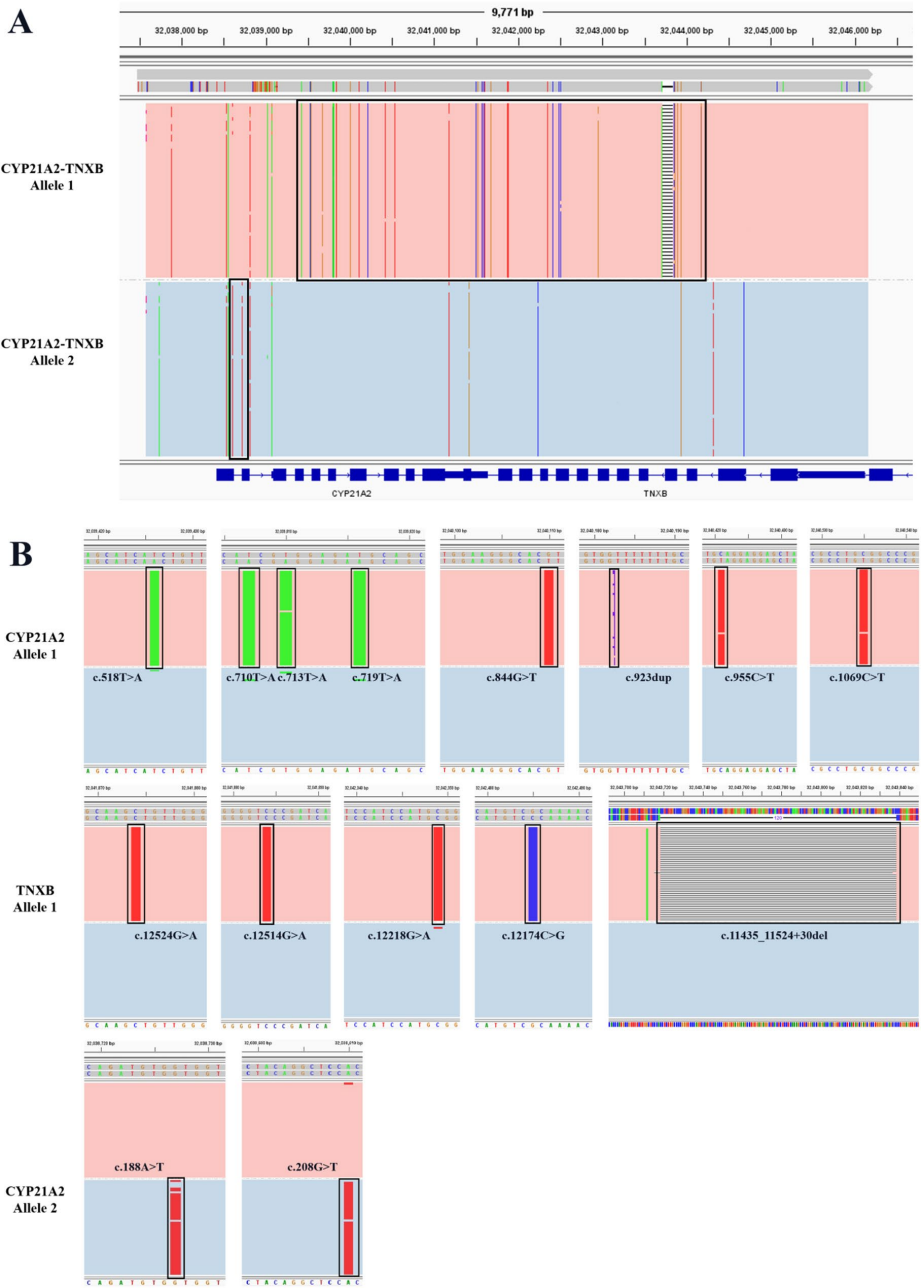
Integrative Genomics Viewer (IGV) plots of long-read sequencing results of patient 1. (**A**) Haplotypes of *CYP21A2-TNXB* genes. The black box in *CYP21A2-TNXB* allele 1 indicates the region of large gene conversion (chr6:32039426–32044184), and each pathogenic variant is amplified in Part B. The black box in *CYP21A2-TNXB* allele 2 indicates the two variants: *CYP21A2*: c.188 A > T and c.208G > T, and they are also amplified in Part B (**B**). The black boxes indicate the specific pathogenic variants on each allele. From top to bottom, and from left to right, in sequence: *CYP21A2*: c.518T > A, c.710T > A, c.713T > A, c.719T > A, c.844G > T, c.923dup, c.955 C > T, and c.1069 C > T; *TNXB*: c.11435_11524 + 30del, c.12524G > A, and c.12218G > A,c.12,174 C > G; *CYP21A2*: c.188 A > T and c.208G > T

The LRS testing revealed a large gene conversion on the *CYP21A2-TNXB* allele 1, specifically, a continuous gene sequence from the exon 4 of the *CYP21A2* gene to the intron 33 of the *TNXB* gene (chr6:32039426–32044184) underwent a large conversion with the corresponding sequence of the *CYP21A1P-TNXA* pseudogene. This resulted in a series of pseudogene-derived variants (*CYP21A2*: c.518T > A, c.710T > A, c.713T > A, c.719T > A, c.844G > T, c.923dup, c.955 C > T, c.1069 C > T; *TNXB*: c.11435_11524 + 30del, c.12524G > A, and c.12218G > A,c.12174 C > G) being detected in the *CYP21A2-TNXB* gene (Fig. 3). On the *CYP21A2-TNXB* allele 2, c.188 A > T and c.208G > T variants were detected in *CYP21A2*, without *TNXB* pathogenic variants (Fig. 3).

As previously mentioned, the c.11435_11524 + 30del variant on exon 35 of the *TNXB* allele 1 rendered the gene nonfunctional and decreased expression levels because of haploinsufficiency, and association with the EDS phenotype. Variants on *CYP21A2* allele 1 resulted in complete loss of function, and the c.188 A > T and c.208G > T variants in *CYP21A2* allele 2 were associated with the NC and SV types of 21-OHD phenotype, respectively [19]. This patient displayed the EDS phenotype and SV-type 21-OHD phenotype, which was consistent with the genotype, and was diagnosed with CAH-X syndrome.

**Fig. 4:**
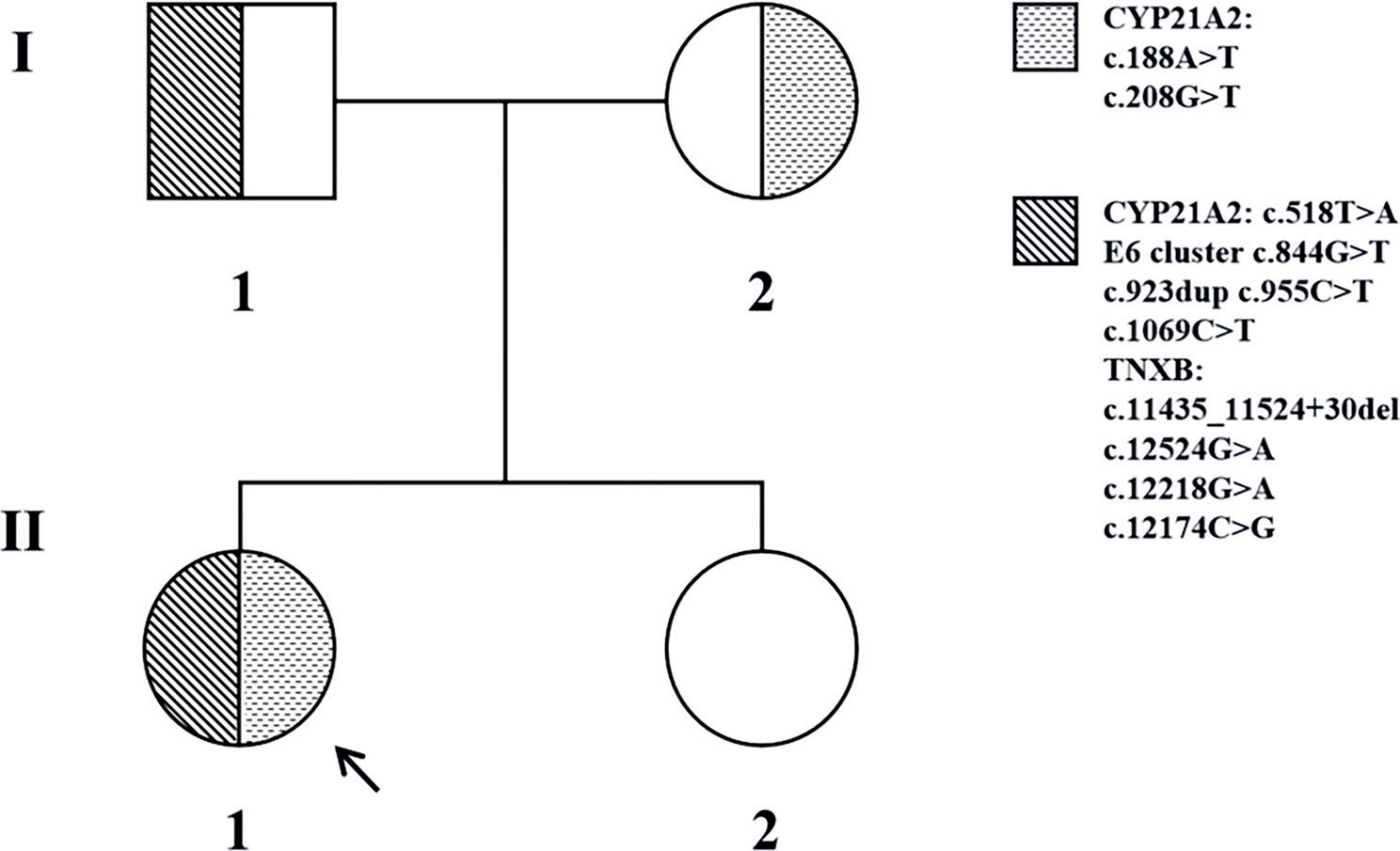
The genetic pedigree of the Patient 1. The arrow indicates the patient

Furthermore, we conducted clinical data collection and genetic testing on the patient’s family members. Her family members, including her mother, father, and a 3-year-old sister, were all in good health, without a medical history of 21-OHD, and did not exhibit manifestations related to EDS, and both had a Beighton score of 0. Genetic testing revealed that, one of the CYP21A2-TNXB alleles in the her father carried a series of variants, that is, *CYP21A2*: c.518T > A, c.710T > A, c.713T > A, c.719T > A, c.844G > T, c.923dup, c.955 C > T, and c.1069 C > T, *TNXB*: c.11435_11524 + 30del, c.12524G > A, c.12218G > A, and c.12,174 C > G, as well as the series of variants that detected on the patient’s *CYP21A2-TNXB* allele 1, while the other *CYP21A2-TNXB* allele in her father did not carry any pathogenic variants. This indicates that the novel CAH-X genotype resulting from large gene conversion in the patient was inherited from her father. Additionally, it was revealed that one of the *CYP21A2-TNXB* alleles in her mother carried the CYP21A2 c.188 A > T and c.208G > T variants, and the other *CYP21A2-TNXB* allele carries no pathogenic variants. The patient’s 3-year-old sister had not been found to carry pathogenic variants. The genetic pedigree of the patient was illustrated in Fig. 4.

### Patient 2

A 9-year-old girl showed signs of abnormalities in her external genitalia shortly after birth, including obvious pigmentation and enlargement of the clitoris. She did not show symptoms of salt-wasting but exhibited significantly elevated serum levels of 17-OHP and T, with normal levels of sodium and potassium. As a result, she was diagnosed with an SV-type of 21-OHD.

In the assessment of the EDS phenotype, her Beighton score was 5 points with bilateral positive for hyperextension of the fifth fingers (Fig. 2C), bilateral positive for elbows hyperextension (Fig. 2D), and positive for the palm-to-floor test. However, she did not exhibit any other symptoms related to EDS, such as skin hyperextensibility or soft skin, as well as any cardiac abnormalities.

**Fig. 5:**
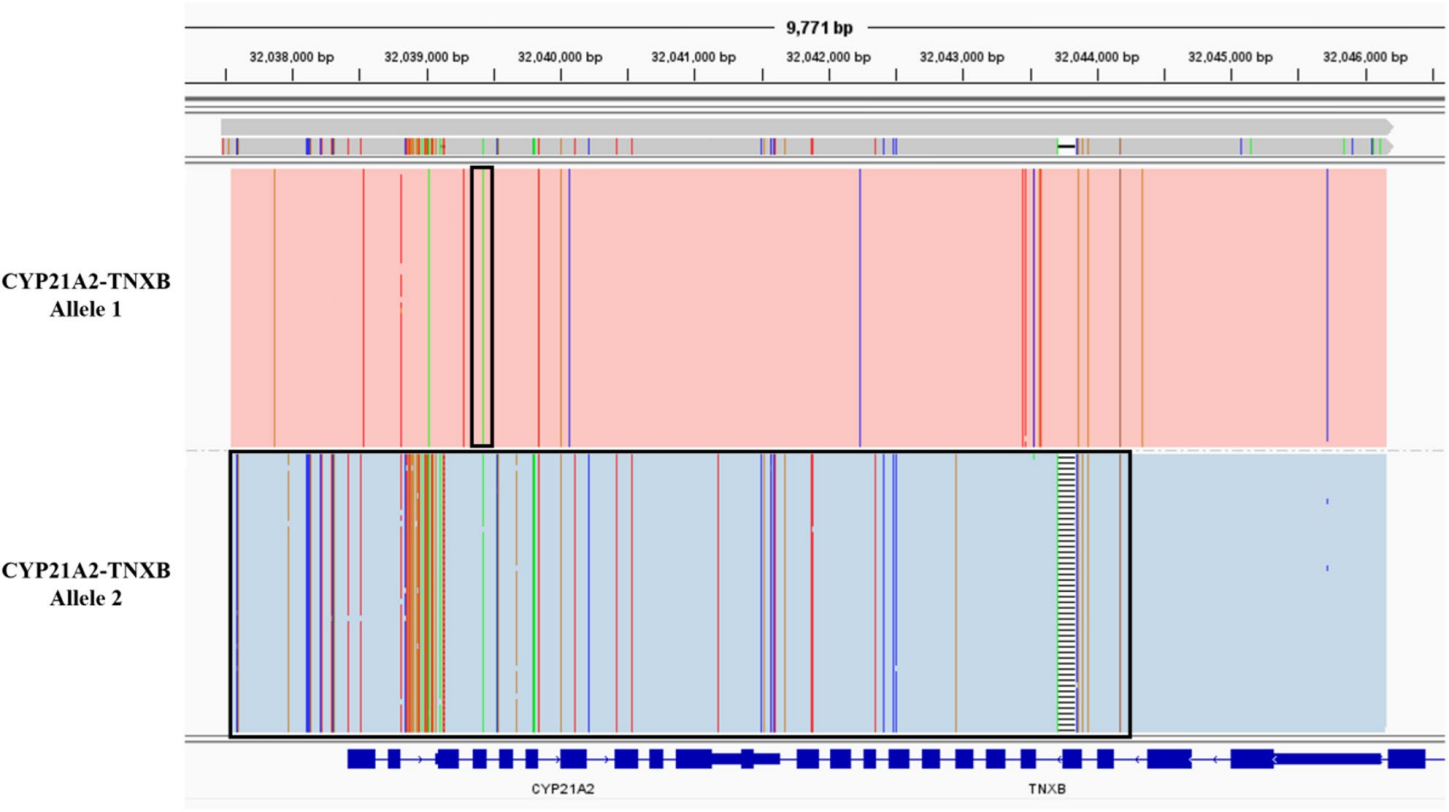
Intergrative Genomics Viewer (IGV) plots of long-read sequencing results of patient 2. The black box in *CYP21A2-TNXB* allele 1 indicates the variant: *CYP21A2*: c.518T > A. The black box in *CYP21A2-TNXB* allele 2 indicates the *CYP21A1P-TNXA* pseudogene sequence regions located on the *TNXA/TNXB*_CH-1 chimeric gene structure

Furthermore, LRS testing revealed that the structure of *CYP21A2-TNXB* allele 2 consists of a *TNXA/TNXB*_CH-1 chimeric gene (Fig. 5). On the *CYP21A2-TNXB* allele 1, the SV type‒associated variant c.518T > A was detected in *CYP21A2*, without *TNXB* pathogenic variants (Fig. 5). These assessments showed that the patient was diagnosed with CAH-X syndrome.

## Discussion

**Fig. 6:**
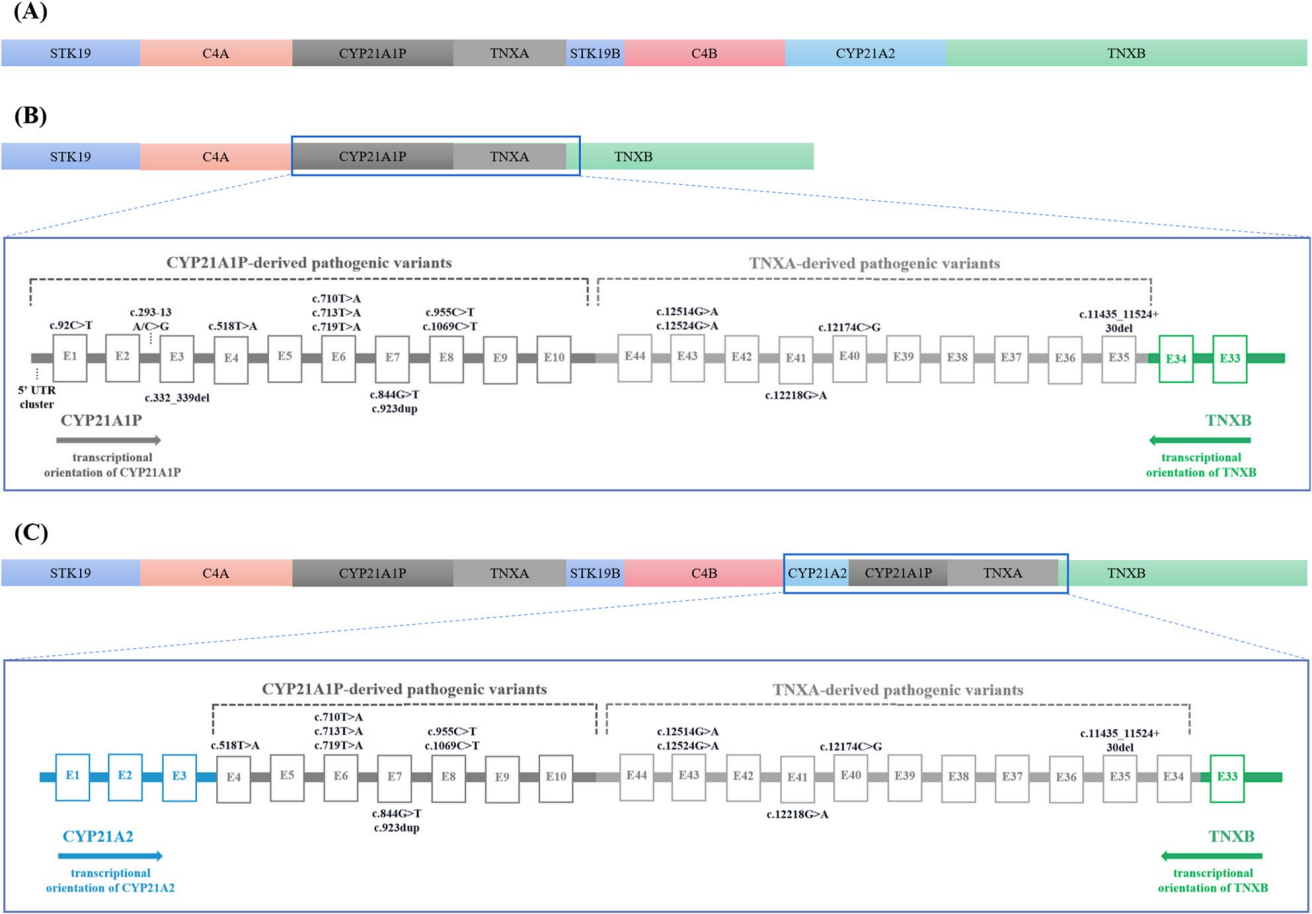
The RCCX haplotype and the *CYP21A2-TNXB* gene structure of the novel CAH-X genotype in patient 1, compared with other RCCX haplotypes. (**A**) Wild-type bimodular RCCX haplotype. (**B**) Chimeric *TNXA/TNXB* gene in monomodular RCCX haplotype, characterized by 30-kb deletion. The diagram displayed in the indigo box illustrates the structure of the CAH-X CH-1 chimeric gene. (**C**) The novel CAH-X genotype that were found in patient 1, which resulted from large gene conversion involving a continuous gene sequence from the exon 4 of the *CYP21A2* gene to the intron 33 of the *TNXB* gene

This study used LRS testing for the genetic diagnosis of CAH-X syndrome. Previous studies implicated that CAH-X syndrome cases were primarily caused by the chimeric *TNXA*/*TNXB* genes that are characterized by the 30-kb deletions [4–9]. In the RCCX module, gene conversion events between pseudogenes and functional genes were more common than misalignment events that caused 30-kb deletions. Gene conversion can take place between *CYP21A2* and *CYP21A1P*, as well as between *TNXB* and *TNXA*. However, there have been no reports on CAH-X syndrome resulting from gene conversion to date, and we identified a novel CAH-X genotype caused by a large gene conversion for the first time. The LRS test results for patient 1 revealed a large sequence that spanned the *CYP21A2* and *TNXB* genes, which underwent conversion with the *CYP21A1P*-*TNXA* pseudogene. The *TNXB* gene was found to be contiguous with the *CYP21A2* gene, without a 30-kb deletion, indicating a novel genotype (Fig. 6). Overall, this novel CAH-X genotype resulting from large gene conversion is functionally similar to the CAH-X CH-1 chimera with some structural differences. In the *CYP21A2* gene region, the novel CAH-X genotype retained the sequence of the *CYP21A2* gene with numerous pathogenic variants from *CYP21A1P*, thereby rendering it completely nonfunctional. In contrast, the CAH-X CH-1 chimera loses the entire *CYP21A2* gene because of the 30-kb deletion in the RCCX module. In the *TNXB* gene region, both the novel genotypes and the CAH-X CH-1 chimera retained multiple pathogenic variants from *TNXA*, with the c.11435_11524 + 30del variant on exon 35 resulting in haploinsufficiency. In short, this novel CAH-X genotype leads to complete loss of function of the *CYP21A2* gene and haploinsufficiency of the *TNXB* gene, exhibiting functional characteristics similar to the CAH-X CH-1 chimera.

Further, this study highlights the potential presence of a category of CAH-X genotypes associated with gene conversions. Theoretically, these genotypes can be classified into 2 subcategories, that is, large conversions on the continuous sequences of *CYP21A2-TNXB* genes or microconversions occurring simultaneously at non-contiguous loci of *CYP21A2* and *TNXB* genes. Given the high frequency of gene conversion events in the RCCX module, this study speculated that the CAH-X syndrome caused by gene conversion events may not be rare, requiring urgent additional research.

The clinical manifestations of EDS in patients with CAH-X syndrome are complex and diverse. As per the literature, the clinical manifestations are summarized as follows [3–8]: (1) musculoskeletal system – GJH, recurrent joint dislocations, chronic muscle pain or joint pain, etc.; (2) integumentary system – skin hyperextensibility, velvety skin, atrophic scars, unexplained skin striae, fragile skin, susceptibility to bruising, delayed wound healing, etc.; (3) digestive system – abdominal hernias, gastroesophageal reflux, uvula bifida, rectal prolapse, irritable bowel syndrome, etc.; (4) others – cardiac defects, pes planu, piezogenic papules, scoliosis, etc. The EDS phenotype mediated by haploinsufficiency of the CAH-X CH-1 chimeric gene is generally milder, whereas the EDS phenotypes mediated by dominant negative effects of CAH-X CH-2 and CH-3 are relatively more severe [3–8]. Compared to studies outside of China, which indicated that nearly 100% of 21-OHD patients with CAH-X chimeric genes exhibit mild to severe symptoms related to EDS, research from China showed that this proportion is only 71%. This suggested that the EDS phenotype in Chinese patients with CAH-X syndrome may be comparatively milder [6]. In this study, the clinical manifestations of Patient 1 indicate that the novel CAH-X genotype is associated with a mild EDS phenotype, including mild joint and skin symptoms. As previously discussed, this is quite similar to the CAH-X CH-1 chimera. Additionally, according to reports, family members of patients with CAH-X syndromes, specifically, heterozygous carriers of CAH-X chimera who do not have 21-OHD, typically exhibit only mild EDS symptoms or no symptoms at all [3, 4, 6, 14]. In this study, the father of Patient 1, who is a healthy carrier of this novel CAH-X genotype without 21-OHD, showed no EDS-related symptoms, which aligns with expectations. Overall, the EDS symptoms in the two cases of CAH-X syndrome were both mild, which may be related to the younger age of the patients and the milder EDS phenotype in the Chinese population. Anyhow, longer-term follow-up is needed.

Owing to the complexity of pathogenic genes, the diagnosis of CAH-X syndrome is quite challenging. Conventional Sanger sequencing for 21-OHD results in a missed diagnosis of CAH-X chimeras, unless a specific PCR method for CAH-X chimeras is performed before Sanger sequencing [14, 23]. However, the specific PCR method for CAH-X chimeras is not routinely conducted in molecular diagnostic laboratories [14]. The multiple ligation-dependent probe amplification (MLPA) can identify variations in gene copy number, but lacks methods for detecting the *TNXB* gene region. At present, only a specific MPLA assay (SALSA MLPA Probemix P050CAH, MRC-Holland) can detect the copy number of *TNXB* exon 35 to identify the CAH-X CH-1 chimera, but cannot identify the CAH-X CH-2 or CAH-X CH-3 chimera [14]. NGS is routinely used in clinical practice, but is unsuitable for RCCX modules with pseudogene interference and complex copy number variations [6]. Multiple studies have reported that LRS testing can identify various genotypes of 21-OHD and is more accurate, comprehensive, and efficient compared with other genetic testing methods [16–19]. Our research demonstrated that LRS may be a reliable method for genetic diagnosis of CAH-X syndrome.

Overall, this study conducted LRS testing on 20 pediatric patients with 21-OHD; among them, 2 were diagnosed with CAH-X syndrome, and 1 of the 2 patients exhibited a novel CAH-X genotype resulting from large gene conversion. The results of this study highlight the potential existence of further CAH-X genotypes related to gene conversion and indicate that LRS may be a more reliable method for genetic diagnosis of CAH-X syndrome and aid in the discovery of novel genotypes. In the near future, with reduced costs and simplified workflow in clinical applications, LRS is expected to improve the molecular diagnostic rates for patients with CAH-X syndrome and further expand the genetic spectrum of CAH-X syndrome.

## Data Availability

The data that support the findings of this study are available on request from the corresponding author. The data are not publicly available due to privacy or ethical restrictions.
